# India’s Conditional Cash Transfer Programme (the JSY) to Promote Institutional Birth: Is There an Association between Institutional Birth Proportion and Maternal Mortality?

**DOI:** 10.1371/journal.pone.0067452

**Published:** 2013-06-27

**Authors:** Bharat Randive, Vishal Diwan, Ayesha De Costa

**Affiliations:** 1 Department of Public Health, R. D. Gardi Medical College, Ujjain, India; 2 Epidemiology and Global Health, Department of Public Health and Clinical Medicine, Umea University, Umea, Sweden; 3 Division of Global Health, Department of Public Health Sciences, Karolinska Institutet, Stockholm, Sweden; Tehran University of Medical Sciences, (Islamic Republic of Iran)

## Abstract

**Background:**

India accounts for 19% of global maternal deaths, three-quarters of which come from nine states. In 2005, India launched a conditional cash transfer (CCT) programme, *Janani Suraksha Yojana* (JSY), to reduce maternal mortality ratio (MMR) through promotion of institutional births. JSY is the largest CCT in the world. In the nine states with relatively lower socioeconomic levels, JSY provides a cash incentive to all women on birthing in health institution. The cash incentive is intended to reduce financial barriers to accessing institutional care for delivery. Increased institutional births are expected to reduce MMR. Thus, JSY is expected to (a) increase institutional births and (b) reduce MMR in states with high proportions of institutional births. We examine the association between (a) service uptake, i.e., institutional birth proportions and (b) health outcome, i.e., MMR.

**Method:**

Data from Sample Registration Survey of India were analysed to describe trends in proportion of institutional births before (2005) and during (2006–2010) the implementation of the JSY. Data from Annual Health Survey (2010–2011) for all 284 districts in above- mentioned nine states were analysed to assess relationship between MMR and institutional births.

**Results:**

Proportion of institutional births increased from a pre-programme average of 20% to 49% in 5 years (p<0.05). In bivariate analysis, proportion of institutional births had a small negative correlation with district MMR (r = −0.11).The multivariate regression model did not establish significant association between institutional birth proportions and MMR [CI: −0.10, 0.68].

**Conclusions:**

Our analysis confirmed that JSY succeeded in raising institutional births significantly. However, we were unable to detect a significant association between institutional birth proportion and MMR. This indicates that high institutional birth proportions that JSY has achieved are of themselves inadequate to reduce MMR. Other factors including improved quality of care at institutions are required for intended effect.

## Background

India accounted for 19% of the globally estimated 287 000 maternal deaths in 2010 [1]. Although the level of maternal mortality in India has shown a definite decline over the last decade nationally, the MMR declined by 35% from 327 deaths per 100 000 births in 1999–2001 [2] to 212 in 2007–2009 [3]; the current number of maternal deaths is still unacceptably high. The national MMR in India is an aggregate that conceals wide regional variations. Three large states viz. Kerala, Tamil Nadu, and Maharashtra with MMRs of 81, 97, and 104 per 100 000 births respectively, have already achieved the Millennium Development Goal 5 (MDG 5) target [4]. However, in nine other large states, MMR estimates still range between 258 and 390 [3]. These nine states account for 62% of maternal deaths in India, and 12% of the global burden of maternal mortality [3]. The world’s progress towards the achievement of MDG 5 is largely dependent on maternal mortality reductions in India, more specifically, in these nine Indian states.

Skilled attendance at all births is considered to be the most critical intervention for ensuring safe motherhood [5], [6] and has been accepted as one of the indicators for measuring progress to achieving MDG 5 [7]. Institutional delivery is expected to improve maternal and neonatal outcomes through timely intervention by skilled birth attendants backed by essential infrastructure and strong referral services when needed.

To reduce maternal mortality, the Government of India commissioned nationwide programmes, including the Child Survival and Safe Motherhood Program (1992–1997) [8] that was followed by Phase-1 of the Reproductive and Child Health Programme (RCH-1) (1997–2004) [9]. These programmes aimed at increasing the availability of emergency obstetric care (EmOC) services by enhancing institutional capacities. However during this period (1992–2004) of focus on strengthening institutions and investing in the supply side, maternal health care indicators in India were slow to improve, despite it being a period of substantial economic growth in the country. A major bottleneck identified was the low demand for and uptake of institutional deliveries – the proportion of institutional deliveries during this period showed increase from 26% to 41%; skilled birth attendance increased from 33% to 47%, yet more than half of women continued to deliver at home [10], [11].

Demand–side financing programmes, particularly cash transfer programmes, have emerged recently as newer ways of addressing the chronic problem of underutilisation of health and social services, particularly among vulnerable groups. The PROGRESA programme in Mexico provided cash to families in return for accessing children’s education, health, and nutrition services [12]. Evaluation of the PROGRESA programme showed a significant positive impact on school enrolment and health outcomes [13]. Similarly, the conditional cash transfer (CCT) in Honduras showed that conditional payments to households increased the use and coverage of preventive health care interventions [14]. Evaluation of cash transfer programmes in Nicaragua [15], Colombia [16], and Brazil [17] also demonstrated a similar positive effect. The most common mechanisms that have been employed in these regions to stimulate demand have been CCTs and voucher schemes. The CCT provides monetary incentive to households/individuals on the condition that they utilise specific services.

Given the limited success with supply–side interventions under RCH-1 (1999–2004) in raising the proportions of skilled attendance at births [18] and the growing evidence of the effectiveness of demand–side financing schemes on the utilisation of health services [19], [20], [21] the Indian government, in 2005, launched a nationwide CCT programme known as Janani Suraksha Yojana (JSY) focussed on maternal health. The JSY aims to reduce maternal and neonatal mortality through the promotion of institutional births by providing cash incentives to mothers on giving birth in a health institution. While the outline of the JSY scheme is the same across the country, it has different eligibility criteria and differential cash transfer size in different states, based on provincial proportions of institutional birth at the time the scheme was designed. In the states with high levels of maternal mortality and low levels of institutional delivery(low performing states), the JSY scheme provides a cash incentive of $31 and $22 to rural and urban women respectively, irrespective of socioeconomic status, age, or parity if they give birth in a public or accredited private health facility. In other socioeconomically better-developed states (high-performing states), the cash incentive is about half that paid out in the low performing states and is restricted to the first two live births of women from below the poverty line (BPL) and from scheduled castes (SC) and tribes (ST) [22]. All health facilities pay incentives into the mother’s bank account at the time of discharge from health facility after delivery. To provide mothers more options to choose the place for delivery, in some districts, private health facilities are accredited by district-level authorities based on broad guidelines issued by the health ministry. These guidelines include criteria such as infrastructure and human resources required [23]. Women delivering at private facilities receive JSY benefits only on producing official certification of belonging to a vulnerable group. The Indian CCT scheme is the largest CCT in the world, with 52 million [24] beneficiaries since inception.

The CCT is underpinned by two major assumptions: (1) Financial barriers exist to access institutional care for childbirth. The cash incentive will enable women to overcome these financial barriers to access institutional care for delivery, and (2) increasing institutional births will provide more women access to skilled birth attendance and, therefore, will reduce maternal and neonatal deaths. Thus, the CCT is envisaged to result in (a) increased institutional births and (b) reduced MMR and neonatal mortality in regions with high proportions of institutional births.

Previous evaluations of the Indian CCT, i.e., the JSY, were limited to small geographic areas [25] focussed on processes [26] and/or based on data from early years of the JSY [27] and have limitations owing to unavailability of maternal mortality data at district levels. The Government of India recently set up the Annual Health Survey (AHS) (2010–2011) to capture population-based data on health indicators in these nine states with poor health indicators including high levels of maternal mortality.

In this paper, we (i) report on trends in uptake of institutional births after the initiation of the CCT, and (ii) study the association between institutional birth proportions and the MMR in these nine states using AHS data. There have been calls in the literature to investigate the success of such CCT programmes in low-income settings, with more limited health system capacity [21]. This paper contributes to this body of knowledge; - lessons from this study of the Indian CCT will be valuable in informing policies around demand-side financing in other similar settings.

## Method

### Study Setting

India is federal union of 35 states with distinctly different levels of socioeconomic development. States are further subdivided into smaller administrative units called districts, each with a population of approximately 1.5 million, which is divided into five to ten units called blocks.

**Figure 1:**
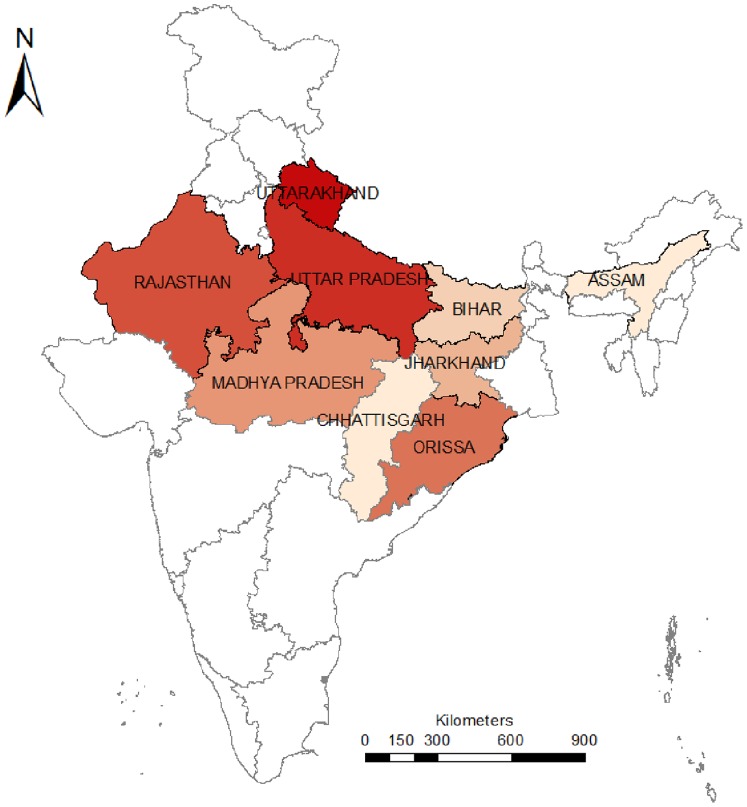
nine study states in India.

This study includes nine large states - Bihar, Uttar Pradesh(UP), Uttarakhand, Madhya Pradesh(MP), Orissa, Rajasthan, Jharkhand, Chhattisgarh, and Assam – that constitute about half of India’s population and account for 62% of her maternal deaths. These nine states are subdivided into 284 districts (see Fig. 1). They have relatively poor socioeconomic indicators; 34%–57% of their populations live below poverty line [28] (based on a defined degree of deprivation) as per national surveys carried out by the Indian government. These nine states have relatively higher MMRs, infant mortality rates (IMR), and birth rates than the national averages of 212/100,000 live births, 50/1000 births, and 22.5/1000 population, respectively. The Government of India has classified these states as ‘high focus states’, implying more focussed attention to and greater allocation of resources towards strengthening the health system [29] in these states.

### Study Design

This study is an analysis of secondary data from two large population-based national surveys viz. Sample Registration Survey and Annual Health Survey conducted by the Government of India.

### Data Sources: The Sample Registration System (SRS) [30]

The SRS is a large demographic survey carried out periodically in India to generate reliable annual estimates of birth rate, death rate and other fertility and mortality indicators at the national and state levels. At present, SRS is operational in all states of India and covers about 7.27 million people in 1.5 million households. The sample unit in rural areas is a village and in urban areas; the sampling unit is a census enumeration block (population from 750 to 1000). The SRS comprises continuous enumeration of births and deaths in selected sample units by resident part-time enumerators, and an independent survey every six months by SRS supervisors. The data obtained by these two independent functionaries are matched. While recording details of every outcome of pregnancy, the enumerators and supervisors are required to enquire about the type of medical attention received by the mother at the time of delivery, including place of delivery.

Proportions of institutional births reported by the SRS between 2005 and 2010 (years) have been used for the analysis in this paper.

#### Annual Health Survey (AHS)

[31]: The AHS is the Government of India’s recent initiative at recording district level health outcomes in the nine ‘high focus’ states. The rationale for the survey was to identify districts requiring special attention as these often miss detection when studying average statistics at the state level. A special feature of the AHS is that it is the first survey in India to provide estimates of the district level mortality.

The first AHS reported in 2010–2011 covered all the districts in the nine states. The AHS adopted a uni-stage stratified simple random sample without replacement except in case of larger villages and surveyed 18 million people in 3.6 million households. The survey collected background information of selected households and information from ever married women aged 15–49 years from these households regarding pregnancy outcome, place of delivery, child immunisation, and breastfeeding, to mention a few, that took place during the reference period (Jan. 2007 to Dec. 2009). Further details of data collection and management procedures are available on the survey website.

Data reported by AHS on district-level MMR, proportion of institutional births, caesarean rate, total fertility rate, and proportion of literate and poor population were used in this paper.

#### Census of india

The Indian national census is conducted every ten years across all (35) states in the country. Each household is visited to collect information on a wide range of demographic and socioeconomic indicators of the household and the individuals. The district level information on level of urbanisation, vulnerable population, and total population from Census 2001 were used as covariates in the analysis presented here.

### Variables

For state-level analysis, the proportion of institutional births, i.e., number of births that took place in government or private health institutions out of the total births, before and during the implementation of the JSY (2005–2010) was used. These data were sourced from the SRS during the period.

For district-level analysis, the main outcome variable was district MMR. Given that maternal mortality is a rare event, the AHS estimated the MMR for a group of three to five geographically contiguous districts. In this analysis, we attributed the estimated MMR to each district in the group.

The main explanatory variable of interest was district level proportion of all institutional births. Other socio-demographic covariates that influence maternal mortality included district-level caesarean rate, total fertility rate, literacy, proportion of households in the lowest 20% quintile of wealth index, proportion of vulnerable (Scheduled cast/tribes) and urban population. A brief description of these variables is given below:

#### Caesarean rate

Percentage of caesarean deliveries out of total deliveries that occurred at government and private institutions in the given period. Caesarean rate is the proxy indicator for availability of comprehensive EmOC services.

#### Total fertility rate

The total fertility rate (TFR) in a specific year is the number of children that would be born to each woman if she were to live to the end of her childbearing years and if the likelihood of her giving birth to children at each age was the currently prevailing age-specific fertility rates.

#### Literacy

The proportion of population in a district with the ability to read and write in any language, expressed as a percentage.

#### Proportion of poor households

Household wealth index was constructed by the AHS at the state level for each of the nine study states using the assets possessed (such as ownership and status of the house) and the facilities availed (such as electricity, toilet) by the households to determine a household’s relative economic status. Thereafter, the households were ranked according to their individual household asset score and then divided into five quintiles with the same number of households in each. In this paper, we used proportion of households in the lowest-income quintile in each district based on assets possessed as an indicator of level of deprivation (poverty) of the respective districts.

#### Proportion of vulnerable (Scheduled cast/tribes) population

The proportion of scheduled caste and tribe persons in the population of each district. Scheduled castes and tribes are those communities that were historically subject to social disadvantage and exclusion. They are accorded special status by the Constitution of India and are recipients of special social benefits as part of a programme of positive affirmation.

#### Urban population

The proportion of the total population in urban areas for every district.

Data for district-level MMR, institutional births, caesarean rate, TFR, literacy, and poor population were sourced from the AHS and while that for vulnerable, urban and total population of district was sourced from the national census 2001.

### Ethics Statement

The study is based on the data available in the public domain for use.

### Analysis

State-level data on proportion of institutional births between the years 2005 and 2010 from the SRS were analysed to describe trends in proportions institutional births before (2005) and during (2006–2010) the implementation of the JSY. A statistical comparison of mean institutional delivery proportions before (2005) and during (2010) the JSY programme was done.

Socio-demographic characteristics of the 284 districts in the nine states are presented. The association of district characteristics and institutional births was first examined separately by simple correlation analysis.

To study the relationship between the proportion of institutional births and MMR, TFR, literacy, and the proportions of poor, urban and vulnerable populations we first used a simple correlation analysis. Subsequently, multiple regression models were developed to assess the effect of change in the district-level proportion of institutional births on the district MMR when other relevant socio- demographic variables were kept constant.

STATA 10 was used for statistical analysis.

## Results

**Figure 2:**
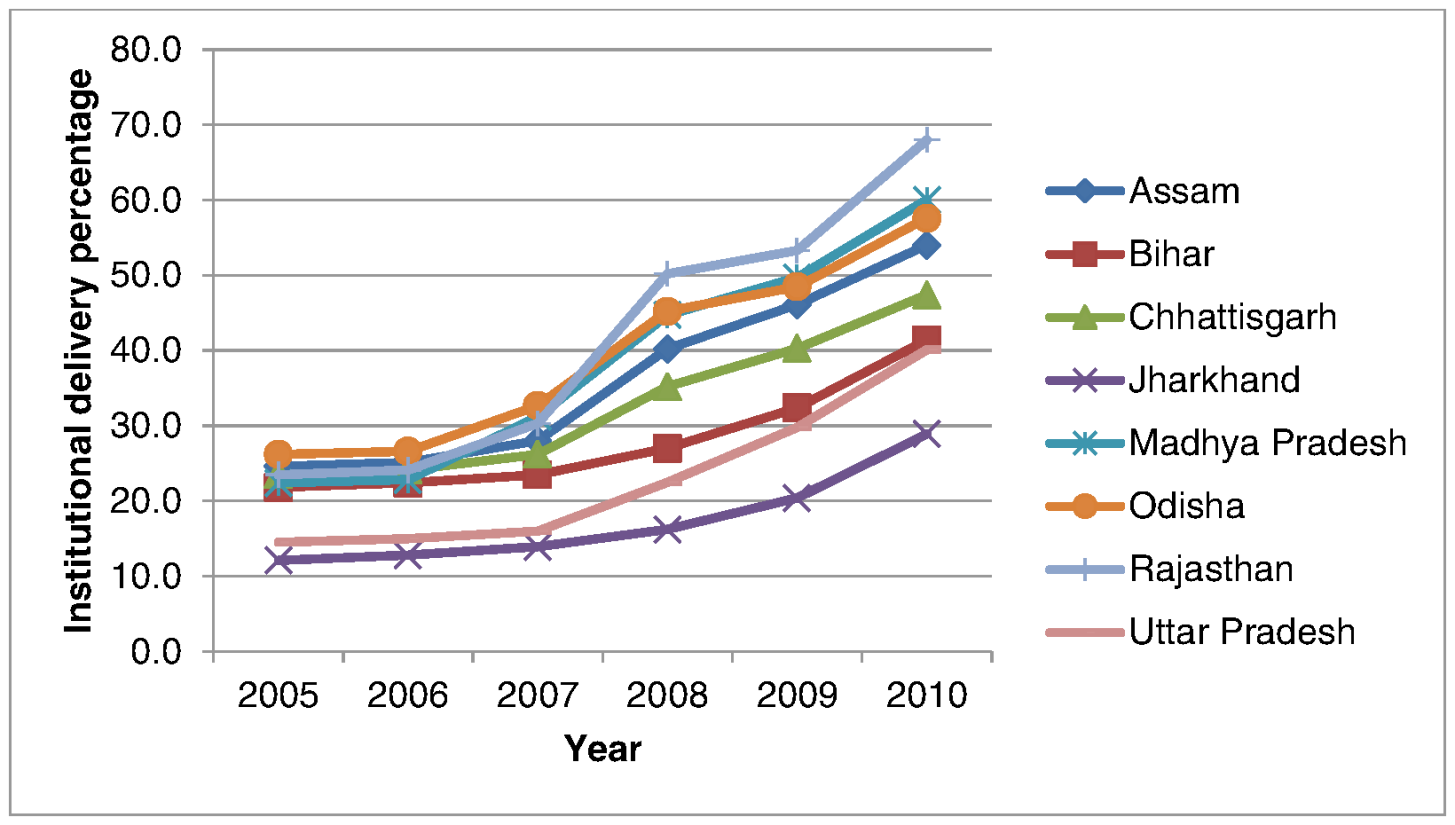
in institutional births.

**Figure 3:**
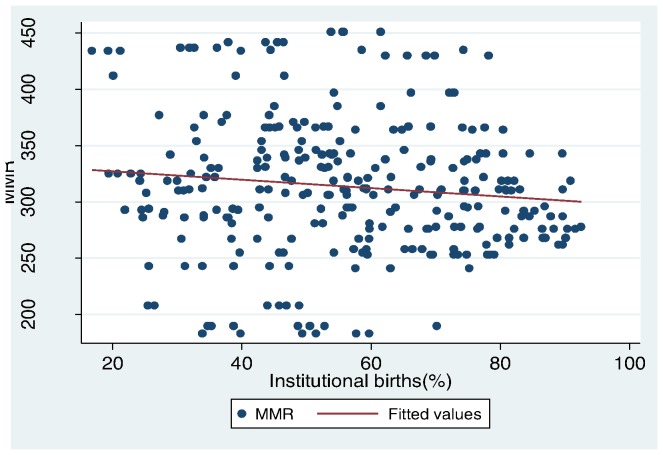
plot of MMR and proportion institutional births.

1. Change in the proportion of institutional births in the nine states since the inception of the JSY programme: The proportion of institutional births increased in the nine states from a pre-programme average of 20% to 49% in the five years (p<0.05). While institutional birth proportions increased across all nine states, the magnitude of the increases varied across states (Fig. 2).
2. Association between institutional birth proportions and MMR in the district:
  - 2.1 *Characteristics of 284 study districts*: District characteristics for the 284 districts are presented in table 1. On average, each district had a population of 1.7 million with varying proportions of poverty, literacy, and urbanisation. The proportion of institutional births ranged from 16.8% to 92.5% (mean 56.2%), demonstrating wide variations in utilisation of this service. The MMR ranges from a minimum of 183 to a maximum of 451.
  - *2.2 Correlation of district characteristics with proportion of institutional births and with MMR:* Table 2 shows the estimated correlation of district characteristics with institutional birth proportions and MMR. Districts with higher fertility rates and higher levels of deprivation had lower institutional births proportions (r = −0.37 and −0.28, respectively); conversely, higher literacy and urbanisation in a district correlated positively with institutional births proportions (r = 0.38 and 0.32, respectively). The proportion of vulnerable population in a district did not show much influence on the uptake of institutional births (r = 0.07). There was no correlation between the proportion of SC/ST populations in the district and institutional birth proportions when these groups were analysed separately (data not shown).
  - Simple correlation between district characteristics and MMR (Table 2) showed that the fertility rate and the proportion of the poor in the population were positively correlated with MMR (r = 0.40 and 0.25, respectively). On the contrary, higher literacy and urbanisation were negatively correlated to MMR (r = −0.34 and −0.18, respectively). The proportion of births in an institution and births by caesarean section (CS), each had a small negative correlation with district MMR (r = −0.11 and −0.19, respectively). A scatter plot of institutional birth proportion and MMR does not show any strong relationship between institutional birth proportions and MMR in the districts (Fig.3).
  - *2.3 Regression analysis:* We built a regression model to explore the association between the proportion of institutional births and MMR. Covariates included are shown in table 3. This model was unable to detect a significant association between institutional birth proportion and MMR [CI: −0.10, 0.68] adjusting for other confounders as shown in table 3. Districts with higher fertility rates or higher proportions of poor population were significantly associated with higher MMR. Conversely districts with high literacy and high urbanisation were associated with lower MMR. Districts with high C-section rates were associated with higher MMR.

**Table 1:** Characteristics of 284 study districts.

District characteristics	Mean
Population(million)	1.7 (0.2,5.2)
Literacy (%)	72.0 (48, 89.8)
Poor households (%)	21.4 (1, 63.5)
Urban population (%)	17.4 (1.2, 80.4)
Vulnerable population (%)	29.3 (5.9, 89.7)
Total fertility rate	3.2 (1.7, 5.9)
Institutional birth proportion	56.2 (16.8, 92.5)
C section rate	5.8 (1.1,19.4)
MMR	313.7 (183, 451)

**Table 2:** Correlation: district characteristics, proportion of institutional births and MMR.

District characteristics	Institutional births (%)	MMR
Institutional birth (%)	1	−0.11
Literacy (%)	0.38	−0.34
Poor households (%)	−0.28	0.25
Urban population (%)	0.32	−0.18
Vulnerable population (%)	0.07	−0.08
Total fertility rate	−0.37	0.40
Caesarean rate	0.40	−0.19

**Table 3:** Regression model assessing correlates of MMR.

Variables	Regression Coefficients(95% CI)
Literacy	−1.44 (−2.61, −0.26)
Poor households	1.10 (0.33, 1.87)
Urban population	−0.62 (−1.29, 0.05)
Vulnerable population	−0.46 (−.96, 0.04)
Total fertility rate	29.7 (15.99, 43.49)
Institutional birth	0.29 (−0.10, 0.68)
Caesarean rate	5.08 (1.84, 8.33)

## Discussion

This report is based on district level estimates of MMR from the AHS from India. The results are important given that the nine states included in the study contribute to 12% of global maternal deaths. Efforts made to reduce maternal mortality in this area will impact global achievement of MDG 5. These results are also relevant for policy makers planning to initiate or expand cash transfers for promotion institutional births in other similar settings.

Our analysis of the nine states indicates a steep rise in institutional birth proportions since the inception of the JSY programme. Although available data do not allow segregation of institutional births into JSY and non-JSY births, a large part of this increase in institutional births is fuelled by the JSY.

Despite the steep rise in institutional births, our analysis was unable to detect a significant association between institutional birth proportions and MMR in the districts.

### 

#### Increase in institutional births

When compared with pre-programme levels, the proportion of institutional births at state level increased two to three times over a period of five years since the programme began. Reports from other large-scale household surveys such as the District Level Household Survey [32] and periodic reports from the health system [33] also show increases in the institutional birth proportions after the implementation of the CCT scheme. A slow rise in institutional births during the RCH-I (1997–2004) and a sharp increase after initiation of the JSY scheme indicates its success in converting a significant proportion of home births into institutional births. While some of these institutional births have definitely occurred outside the JSY i.e., in non- accredited private institutions however this proportion remains marginal. Calculations made from the AHS reports show that an average of 13% of all births and 25% of all institutional births occur in private institutions in the study districts. As women delivering at private institutions accredited for the JSY receive the cash benefit, a proportion of institutional births in the private sector (25%) become JSY beneficiaries, leaving only a small proportion of non-JSY institutional births as a whole. Another recent survey report also indicated that in the ‘high focus’ states of MP, Rajasthan, UP, Orissa, and Bihar, the vast majority of all institutional births do occur under the JSY programme: on average, only 12.9% of all institutional births do not occur within the JSY programme [25].

Experience from Nepal has been similar; a cash incentive to women on delivering in a health facility, increased utilisation in maternity services [34]. A review of evidence on demand-side financing for sexual and reproductive health services in low and middle-income countries reports increased utilisation of services as an effect of demand-side financing strategy [35], [36].

Despite the overall increase in institutional births at the state level, within each state there were wide district-level variations that were associated with background socioeconomic characteristics of the districts. Districts with higher literacy and larger urban populations tended to have higher institutional birth proportions, whereas poverty and high fertility rates adversely affected the utilisation of institutional delivery services. As it is known that poor women bear the highest burden of maternal death, the programme still needs to develop mechanisms to reach this most vulnerable group.

#### Association between institutional birth and maternal mortality

Our analysis was not able to detect a significant association between district institutional birth proportion and MMR. While it is possible that we were unable to detect a significant association given the wide confidence intervals around our estimates, it is also possible that there is a limited influence of institutional birth proportions on MMR. Lim et al, in their evaluation of the JSY scheme in India, also reported an inability to detect its effect on maternal mortality at the district level, possibly because of a lack of programme effect or an inadequate sample size to detect the effect [27].

This finding of a lack of association between institutional birth proportions and MMR could suggest the possibility that it is likely that the CCT disproportionately attracts pregnant women without complications to institutions, i.e., women most vulnerable to maternal death are not entering the programme. The mortality among women with complications is likely to be higher if they deliver at home, than if they deliver at institutions, as the latter should have access to a skilled person and EmOC services. There are currently no estimates of the proportion of mothers with complications among home or institutional births. The lack of association could also be related to the poor quality of care offered at institutions to the mothers with complications. Despite the focus on supply-side strengthening in the earlier RCH programmes, there have been recent reports documenting inadequacies in skilled human resources, infrastructure and supplies, which are critical for provision of good-quality care [37].Moreover, as reported by a programme evaluation report of the JSY [38], although all public sector facilities are designated as programme facilities, it is only a rather small number of higher-level facilities that actually have the ability to handle complications. Therefore, women in rural areas reach lower level facilities which are ill-equipped to handle complications. These inadequacies present a challenge to a mother having a safe delivery, even if she reached a facility. Paxton et al found that correlation between proportion of skilled attendance at birth and MMR becomes weaker for developing countries alone than when both developed and developing countries are included together. The correlation further drops for countries having MMR of more than 200. From this analysis the authors conclude that skilled attendance alone is not accountable for higher correlation of skilled attendance and MMR. Countries with low MMR have high proportion of skilled birth attendance and they have high proportions of maternal complications managed with high quality EmOC services [39].

#### The quality of care issues

While our analysis does not deal directly with quality of care under the programme, it is possibly an important explanation for the lack of association between institutional birth proportions and MMR. The available literature is summarised below.

#### Need to ensure skilled attendance in an enabling environment

In promoting institutional births, it was hoped that pregnant women would get skilled attendance at births, and access to appropriate EmOC in the event of complications. The Safe Motherhood Inter-Agency Group has defined *skilled attendance* as a process through which a woman is provided with adequate care during labour, delivery, and the postpartum period [40]. Studies exploring links between skilled attendance at births and maternal mortality suggest a need of 1) a partnership of skilled attendants (health professionals with the skills to provide care for normal and/or complicated deliveries), and 2) an enabling environment of equipment, supplies, drugs and transport for referral [6]. Investigation by Sri B et al on high number of maternal deaths in 2010 in the Barwani district of Madhya Pradesh found a lack of skilled birth attendance, failure to carry out emergency obstetric care in obvious cases of need, and referrals that never resulted in treatment [41]. This report questions the policy of giving cash to pregnant women to deliver in poor quality facilities without first ensuring quality of care and strengthening the facilities to cope with the increased patient loads.

Programme evaluation of the JSY reports that the programme has increased access to delivery by an Auxiliary Nurse Midwife (ANM), nurse, or doctor, but not necessarily to skilled birth attendant (SBA), because most nurses and ANMs who are actually providing services were not trained in the SBA training [38].

The conceptualisation of the JSY programme in India has led to the substitution of the critical component of skilled attendance with a notion of ‘institutional births’ as being equivalent to skilled birth attendance, and, therefore, this as a condition to be met in order to receive the cash benefit from the JSY. Evidence seems to now indicate that the assumption that institutional birth is the same as skilled birth attendance in an enabling environment does not hold [36], [40]. This has resulted in pregnant women arriving in institutions, but this in itself is not necessarily giving them access to skilled attendance.

#### Need to address other non-financial access barriers

The JSY has raised the uptake of institutional births, yet, in some districts, more than half of women continue to deliver at home. This suggests that even if a cash incentive is able to attract more women to facilities, there are still many for whom; other non-financial barriers operate to reduce the likelihood of an institutional birth. Some of these barriers include the inability to access transportation or the costs involved in doing so, non-perception of birth as a risk event, the social status accorded to women, and poor levels of trust in the public health facilities. Some states initiated emergency transport arrangement, which reduce the transportation barrier; however, the other barriers are more complex and require more structural and social change. An analytical framework by Bart Jacob et al provides guidance on interventions to support structural changes in the health system that can reduce some barriers [42]. Significant positive association of literacy, urbanisation and inverse association of fertility rates and poverty in our regression model seem to suggest that overall socioeconomic development contributes significantly in maternal mortality reduction. Although socioeconomic development is merited, reduction in maternal mortality can be achieved even in countries with poor development indicators. For instance, low income-countries such as Sri Lanka, Thailand, and Maldives have shown that universal access to skilled attendance at birth and EmOC services could reduce maternal mortality drastically [43].

#### Negligence of antenatal and postnatal care

The JSY scheme incentivises pregnant women for utilisation of health facility for intra-partum care, whereas antenatal (ANC) and postnatal care (PNC) is not a prerequisite for cash benefit. While splitting the cash benefit can imply large administrative burdens on the programme, a narrow focus on institutionalising intra-partum care restricts opportunities of averting deaths by early detection of risk pregnancies and treatment of common postnatal complications like puerperal sepsis.

### Limitations

AHS estimates MMR for groups of three to five geographically contiguous districts. These pooled estimates were attributed to individual districts during our analysis. Institutional birth proportions are assumed to be JSY deliveries in this analysis, although a small proportion as reported above are non-JSY institutional births. MMR estimates are based on community-level surveys; no information is available on what proportion of deaths occurred within the programme or outside of it.

#### Residual confounding

The results of the study should be interpreted with caution because the association between institutional birth proportions and maternal mortality can be confounded by other known and unknown confounders. Examples of these could be road network in the district, a measure of overall quality of care in the district, emergency transport services available in the district, etc. Some confounders such as cultural practices, awareness about need of and availability of health care services, etc. will influence maternal mortality via access to institutional birth. Variables used in the analysis (e.g. poverty, literacy, urbanisation) serve as proxies for these factors. Factors that determine the quality of care provided in the facilities could have contributed to varying levels of maternal mortality, but in the absence of precise data on quality of care provided in study districts, this study is unable to control the confounding effect of district-level variation in quality of care.

We acknowledge that residual confounding is likely to be present. However, despite the limitation of this ecological study, it explores the association between district-level variation in uptake of institutional births and maternal mortality using available data at the smallest unit of analysis (district) during the implementation of the JSY; it makes an important contribution.

The counter intuitive finding of an association between caesarean rates and MMR could possibly be because confounding factors were not taken into account in the model. It also suggests further exploration of appropriateness and quality of caesarean and post-operative care provided. It could be possible that the sudden rise in institutional deliveries resulted into overcrowding in facilities and quality of care in operation theatres or in postnatal wards was compromised resulting in more deaths. Another possibility could be the higher rates of caesarean in private hospitals, exposing more women to higher risk. In the 284 study districts, the median caesarean rates for public and private sectors reported by the AHS were 5% and 28%, respectively. When we conducted a regression analysis using a stratified caesarean rate in public and private facilities it showed that caesarean rate in private facilities, but not in public facilities, were significantly associated with higher mortality. This needs to be explored more. Bertan et al, in their analysis of global and regional estimates of caesarean rate show that although caesarean rates below 15% are associated with lower maternal mortality; higher rates are predominantly correlated with higher maternal mortality [44].

### Conclusions

We were unable to detect a significant association between the proportion of institutional births and the MMR at the district level, though other indicators of overall development such as literacy showed a significant association with reduction in the MMR. Although the JSY succeeded in raising institutional birth proportions significantly; the same has not translated into significant reduction in the MMR. It is likely that a weak supply side has led to a situation in which increased access to institutional birth has not resulted in reduction in maternal deaths, as mothers are not receiving appropriate or adequate care. It is also possible that the JSY failed to draw mothers with life-threatening complications into institutions, resulting in most of such women continuing to deliver at home, contributing to persistent maternal mortality. Further studies are required to examine the extent to which the JSY increased access to institutional care among mothers with complications. Moreover, to translate the JSY gains in institutional delivery coverage into reduced mortality outcomes, it is important to ensure that all women accessing an institution for delivery receive good quality obstetric care.
